# Single‐cell transcriptomic analysis of small and large wounds reveals the distinct spatial organization of regenerative fibroblasts

**DOI:** 10.1111/exd.14244

**Published:** 2020-12-07

**Authors:** Quan M. Phan, Sarthak Sinha, Jeff Biernaskie, Ryan R. Driskell

**Affiliations:** ^1^ School of Molecular Biosciences Washington State University Pullman WA USA; ^2^ Department of Comparative Biology and Experimental Medicine Faculty of Veterinary Medicine University of Calgary Calgary AB Canada; ^3^ Department of Surgery, Cumming School of Medicine Alberta Children's Hospital Research Institute Hotchkiss Brain Institute University of Calgary Calgary AB Canada; ^4^ Center for Reproductive Biology Washington State University Pullman WA USA

**Keywords:** dermal papilla, fibroblast heterogeneity, scRNA‐seq, web resource

## Abstract

Wound‐induced hair follicle neogenesis (WIHN) has been an important model to study hair follicle regeneration during wound repair. However, the cellular and molecular components of the dermis that make large wounds more regenerative are not fully understood. Here, we compare and contrast recently published scRNA‐seq data of small scarring wounds to wounds that regenerate in hope to elucidate the role of fibroblasts lineages in WIHN. Our analysis revealed an over‐representation of the newly identified upper wound fibroblasts in regenerative wound conditions, which express the retinoic acid binding protein Crabp1. This regenerative cell type shares a similar gene signature to the murine papillary fibroblast lineage, which are necessary to support hair follicle morphogenesis and homeostasis. RNA velocity analysis comparing scarring and regenerating wounds revealed the divergent trajectories towards upper and lower wound fibroblasts and that the upper populations were closely associated with the specialized dermal papilla. We also provide analyses and explanation reconciling the inconsistency between the histological lineage tracing and the scRNA‐seq data from recent reports investigating large wounds. Finally, we performed a computational test to map the spatial location of upper wound fibroblasts in large wounds which revealed that upper peripheral fibroblasts might harbour equivalent regenerative competence as those in the centre. Overall, our scRNA‐seq reanalysis combining multiple samples suggests that upper wound fibroblasts are required for hair follicle regeneration and that papillary fibroblasts may migrate from the wound periphery to the centre during wound re‐epithelialization. Moreover, data from this publication are made available on our searchable web resource: https://skinregeneration.org/.

## 1 INTRODUCTION

Fibroblast heterogeneity is an important and emerging area of research in skin biology.^1^ These cells perform diverse functions in the dermis and hypodermis of the skin.^1^, ^2^ The fibroblast subtypes that are closest to the epidermis are called papillary fibroblasts and are easy to observe histologically in neonatal mouse skin and in human skin.^3^, ^4^ In mice and in humans, there is an age‐related reduction of the papillary region which may play a role in the decreased wound‐healing abilities associated with aging.^5^, ^6^ Furthermore, the papillary fibroblast lineage in murine skin has been shown to be required to regenerate hair follicles as they can differentiate into dermal papilla.^3^ Dermal papillae are another fibroblast subtype that resides at the base of the hair follicle and are necessary for their formation and maintenance.^7^ Consequently, the proportion of papillary fibroblasts within a wound can affect the regenerative outcome. Reticular fibroblast subtypes reside in the reticular dermis, hypodermis and fascia.^3^, ^8^ They produce the extra‐cellular matrix (ECM) of the dermis and possess a pool of progenitors for adipocytes.^9^, ^10^ Reticular fibroblasts do not differentiate into dermal papilla and produce the ECM of the scar.^3^, ^8^ Harnessing the functions of different fibroblast lineages during wound healing is an important step to achieving functional skin regeneration.

The development of scRNA‐seq has provided unprecedented insights into the cellular heterogeneity of different tissues, including the ability to compare fibroblasts between wounds that scar and those that regenerate with hair follicles.^11^, ^12^, ^13^, ^14^ These comparisons of regenerating and scarring wounds using scRNA‐seq have mapped diverse populations of fibroblast within the wound beds but have not fully understood how they can differentiate into dermal papilla to support hair follicle reformation. However, two recent publications investigating the differences between scarring and regenerating wounds by scRNA‐seq now provide a foundational data set to investigate the ability to regenerate.^15^, ^16^

The genetic markers for cellular clusters in scRNA‐seq experiments have recently been translated into a spatial map by histological analysis.^13^, ^17^ These experiments have allowed for the use of fibroblast markers to identify the spatial location of a fibroblast subtype histologically or computationally. For example, Crabp1 expression in wound fibroblasts marks the cells closest to the upper region of the wound bed.^13^ These are referred to as upper wound fibroblasts. Fibroblasts that express Mest and Plac8 are represented in the lower regions of the wound and are referred to as lower wound fibroblasts.^13^, ^17^ The association of scRNA‐seq gene expression and spatial location will provide additional insights into the molecular and cellular mechanisms of fibroblasts heterogeneity during the wound‐healing process.

It has been recently shown that *Hypermethylated in cancer* (Hic1) transgenic CreERT2 mice can label both papillary and reticular fibroblasts at varying efficiencies.^15^ Histological analysis of lineage traced Hic1‐labelled cells revealed high contribution to regenerating dermal papilla in WIHN. However, scRNA‐seq analysis indicated low contribution of Hic1CreER‐tdTomato cells to regeneration‐competent fibroblasts.

Here, we perform a detailed analysis to address this inconsistency and provide an explanation for these results. We also reanalysed three recently published scRNA‐seq data of scarring and regenerative wounds to compare different fibroblast subtypes. In both conditions, we observed the diverging differentiation trajectories between upper and lower wound fibroblasts. Moreover, our analysis highlighted the striking difference in the relative abundance of upper wound fibroblasts between regenerating and scarring wounds. Finally, we performed a computational test to investigate the regenerative competence of upper wound fibroblasts, which unexpectedly revealed that the wound periphery also possesses the equal regenerative potential after the re‐epithelization of large wounds.

## 2 METHODS

### 2.1 Abbasi et al., 2020 data

Generation of scRNA‐seq data (GSE108677) is described.^15^ In short, cells were isolated from large full‐thickness wounds of Hic1‐tdTomato mice treated with tamoxifen at P4‐5. In addition, collected cells were flow sorted for viability and tdTomato expression. In this manuscript, we utilized data from four different 10× Genomics libraries: (1) large wound centre at D14 and tdTomato positive, (2) large wound centre at D14 and tdTomato negative, (3) large wound periphery at D14 and tdTomato positive and (4) large wound periphery at D14 and tdTomato negative. scRNA‐seq BAM files are downloaded from SRA database. BAM files are processed by Velocyto.py (0.17.15) to generate loom files for downstream RNA velocity analysis. A custom reference genome was built using the *cellranger mkref* by adding the tdTomato transgene sequence to FASTA and GTF files of a prebuilt mm10 reference.

### 2.2 Lim et al., 2018 data

We obtained FASTQ files from the single‐cell RNA‐sequencing data (GSE112671) recently published in Lim et al,^11^ which describes the preparation of samples for sequencing. In short, cells from the dermis of skin wounds were collected from Tet‐treated *SM22‐rtTA; tetO‐Cre* and *R26‐SmoM2/Tomato* (*SM22‐SmoM2*) and *SM22‐rtTA; tetO‐Cre; R26‐Tomato* (control) mice at 12 days postwound. Tomato^+^ cells were sorted and processed for library preparation using 10× Genomics Single‐Cell 3′ v2 kit. Libraries were sequenced on Illumina HiSeq 4000 with one full lane per sample. FASTQ files were generated using Cellranger mkfastq. The Cellranger v3.0.2 pipeline was used to align FASTQ files to the *mm10* reference genome and to generate the output files. The output files were run through Velocyto.py (0.17.15) to produce .loom files for downstream and RNA velocity analysis.

### 2.3 Phan et al., 2020 data

Generation of scRNA‐seq data (GSE153596) was described in Phan et al., 2020. Cells were isolated from small (2 mm) wounds at 7 days postwound from wild‐type P2 and P21 mice (*n* = 3). A total of 6 libraries were generated using 10× Genomics 3′ Single‐Cell Gene expression V2 kit. Libraries were sequenced on Illumina HiSeq 4000 with one full lane per sample. Cellranger v3.0.2 pipeline was used to process fastq files and generated output files. The output files were run through Velocyto.py (0.17.15) to produce .loom files for downstream and RNA velocity analysis. In this manuscript, we used all three libraries from P2 wounds and one library (P21_3) from P21 wounds.

### 2.4 scRNA‐seq data analysis

Loom files were analysed using SCANPY (1.5.1)^18^ and scVelo (0.2.1)^19^ following our analysis pipeline (https://github.com/DriskellLab/Hic1‐Lineage‐reanalysis‐Abassi‐et‐al.‐2020). In short, we filtered out cells with less than 200 genes and genes expressed in less than 3 cells. Number of counts per cell was normalized to 10,000 reads per cell. Data were log‐transformed and regressed. Neighbourhood graph of cells was computed using 40 principal components and *n* neighbours = 10. Uniform Manifold Approximation and Projection (UMAP)^20^ was used for dimension reduction, and data were presented as 2D Map. Cell clusters were defined by Leiden clustering.^21^ For scVelo analysis, we performed both stochastic and dynamical models, which resulted in similar outcomes. In this manuscript, the Stochastic model was presented.

## 3 RESULTS

### 3.1 Comparative scRNA‐seq analysis of scarring and regenerating wounds

**Figure 1:**
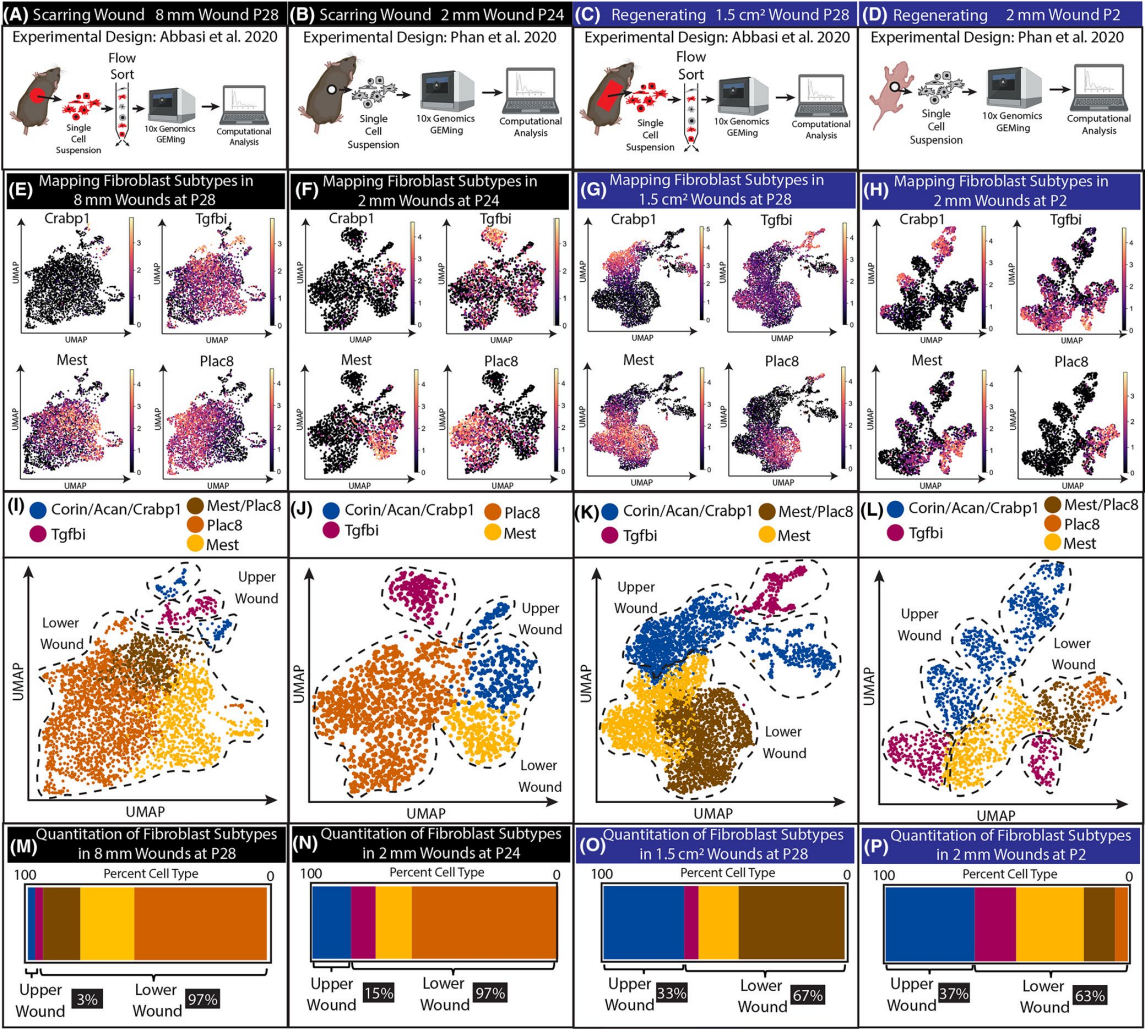
Regenerating wounds contain a higher proportion of upper wound fibroblasts compared with scarring wounds. (A‐D) Experimental design to generate scRNA data from Phan et al., 2020 and Abbasi et al., 2020. (E‐H) Identifying the clusters in UMAPs utilizing upper wound marker Crabp1, and lower wound markers Mest and Plac8. Tgfbi cluster is a newly identified fibroblast cluster in wounds. (I‐L) UMAP of upper, lower and Tgfbi clusters in scarring and regenerative wounds. (M‐P) Quantification of cells within upper, lower and Tgfbi clusters in scarring and regenerative wounds

To investigate differences between scarring and regenerative wound repair, we performed a scRNA‐seq analysis from fibroblasts of two scarring wounds and two types of wounds that regenerate hair follicles (Figure 1). We utilized previously published data from Abbasi et al., 2020 and Phan et al., 2020.^15^, ^16^ scRNA‐seq experiments performed in Phan et al. sequenced all of the cells from single‐cell suspensions of scarring and regenerating wounds. On the other hand, scRNA‐seq experiments in Abbasi et al. utilized here sorted live cells from single‐cell suspensions of small (8 mm circular) and large (1.5 cm^2^ rectangular) wounds.^15^ In addition, lineage tracing of tdTomato labelled Hic1 cells was sorted to generate 10× genomics library (Figure 1A‐D; Figure 3A; Figure S1).

Fibroblast heterogeneity of the wound environment is beginning to be mapped histologically in comparison with scRNA‐seq data.^13^, ^17^ We utilized these mapping strategies to construct fibroblast atlases of the wound environments. To compare and contrast the proportion of upper and lower fibroblasts in wounds between scarring and regeneration, we overlayed upper wound fibroblast markers Crabp1 and lower fibroblast markers Mest and Plac8 on UMAPS generated from each condition (Figure 1E‐H). Furthermore, we also used Corin and Acan (data not shown), which are dermal papilla and dermal sheath‐specific markers for cluster identification. These expression profiles were used to determine which Leiden clusters were associated with upper and lower wound fibroblasts (data not shown; see Github page in Methods) (Figure 1E‐H). We were able to identify a distinct population that expressed high levels of Tgfbi. This Tgfbi high population has overlapping expression with other lower lineage markings. Our reanalysis further confirmed the heterogeneity of fibroblasts detected in both scarring and regenerating wounds.

Furthermore, computational mapping of fibroblast heterogeneity in wounds revealed that scarring wounds have a high proportion of lower wound fibroblasts, while regenerating wounds contained a higher proportion of upper wound fibroblasts (Figure 1I‐L). We quantified the percentage of upper and lower wound fibroblasts present in the Leiden clustering within each wounding condition (Figure 1M‐P). Wounds that regenerate had a consistently higher relative proportion (~33%–37%) of upper wound fibroblasts compare to scarring wound. We conclude that one of the main characteristics of regenerating wounds is the higher proportion of upper wound fibroblasts.

### 3.2 Upper and lower wound fibroblasts have distinct differentiation trajectories in RNA Velocity analysis

To further investigate the dynamic nature of fibroblast heterogeneity in wounds, we performed RNA velocity analysis. RNA velocity estimates the future state of individual cells by calculating the ratio of unspliced and spliced mRNAs to predict lineage trajectories within scRNA‐seq data.^19^, ^22^ We utilized scVelo, an integrated package for SCANPY, to perform this analysis on all four conditions (Methods).

**Figure 2:**
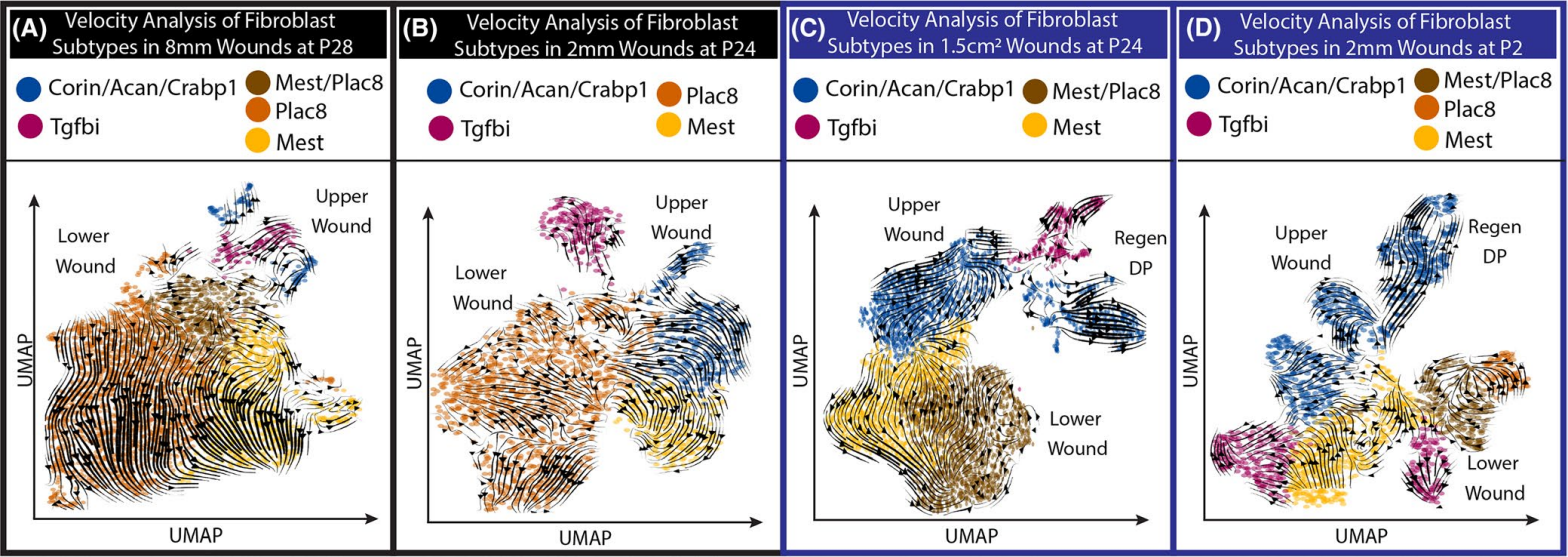
RNA velocity reveals distinct trajectories for upper and lower wound fibroblasts in both scarring and regenerative wounds. (A‐D) Stochastic model of RNA velocity analysis from scarring and regenerative wounds

Upper wound fibroblasts, which express Crabp1, are located closer to the wound epithelium and are thought to be the source of regenerative potential of WIHN. Our velocity analysis revealed that in scarring and regenerative wounds, upper wound fibroblast trajectories project away from lower wound fibroblasts (Figure 2A‐D). Upper wound fibroblasts generally projected trajectories that differentiate away from lower wound fibroblasts. In contrast, lower wound fibroblasts projected trajectories that differentiate away from upper wound fibroblasts (Figure 2A‐D). This result highlighted the divergent trajectories of fibroblasts heterogeneity during the wound‐healing process.

While scarring and regenerative wounds differed in the proportion of fibroblast subtypes, the velocity analysis of these differing wounds revealed similarities in how upper and lower wounds acted across wound types. Importantly, our reanalysis of scarring and regenerative tdTomato cell types in wounds with activated Sonic Hedgehog^11^ reveals similar conclusions (Figure S1; Figure S2; Table S1). The upper Crabp1 fibroblasts were stimulated to differentiate into dermal papilla cells with Shh activation (Figure S2A). We infer that upper and lower wound fibroblasts have distinct differentiation trajectories in the wound.

### 3.3 scRNA‐seq analysis indicates that Hic1 reticular lineage fibroblasts may not contribute to the dermal papilla of WIHN

**Figure 3:**
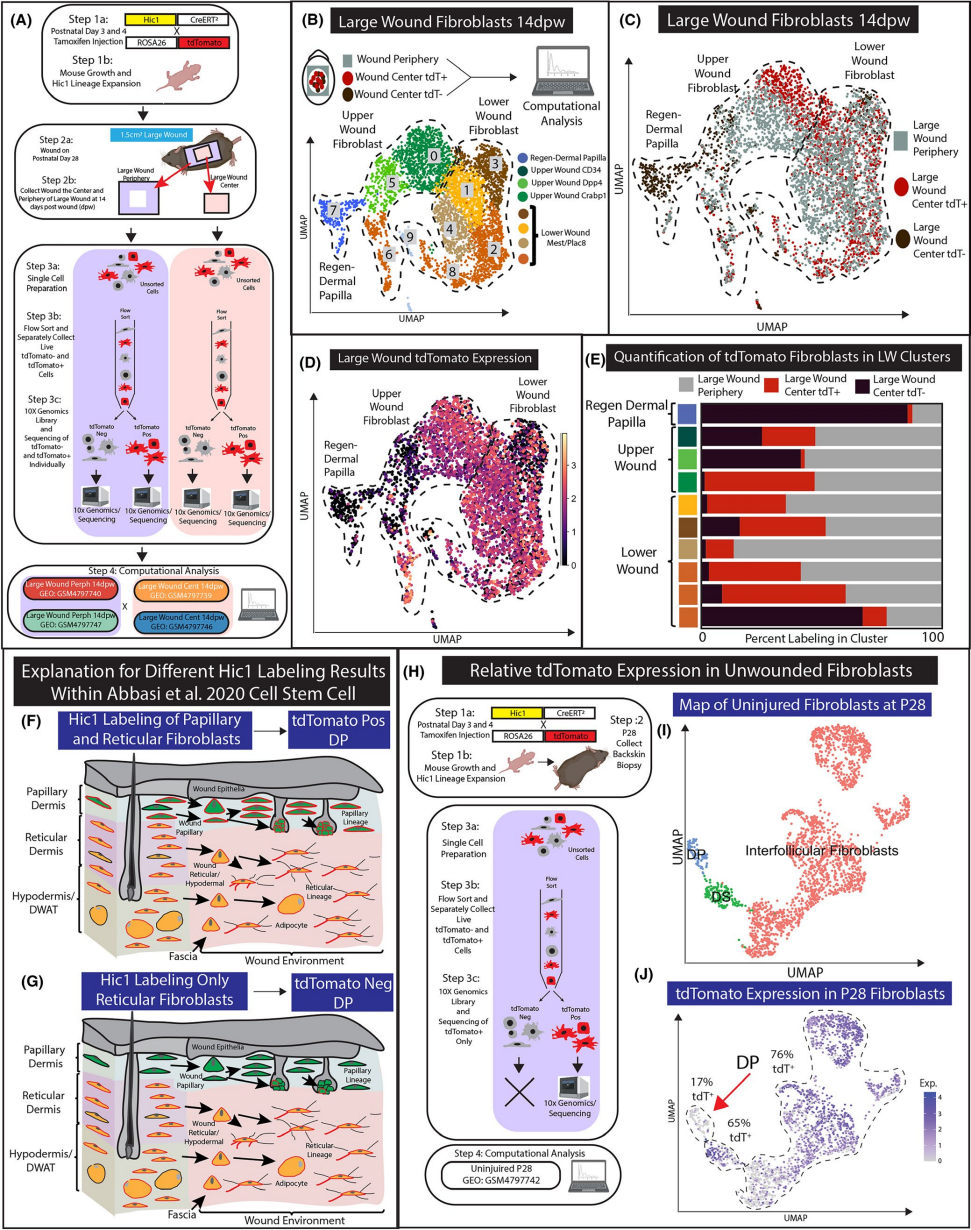
Analysis of Hic1CreERt2‐labelled tdTomato expression in scRNA‐seq data of large wound fibroblast populations. (A) Schematic of experimental design. (B) Recomputed UMAP analysis of all large wound fibroblasts aligned with a genome containing tdTomato. Both wound periphery files (LWP 14dpw Pos and Neg) are coloured grey. (C) UMAP projection colour coded by 10× Genomics libraries of large wounds. (D) UMAP of large wounds with tdTomato expression. (E) Quantification of the number of cells within the Leiden cluster by 10× Genomic library. (F‐G) A proposed explanation for inconsistent results between Hic1CreERT2 histological lineage tracing (F) and scRNA‐seq data (G). (H‐J) An alternative explanation for the lack of tdTomato‐positive cell scRNA‐seq lineage tracing. UMAP projection of fibroblasts subclustered using original Louvain algorithm and grouped as ‘interfollicular fibroblasts’, ‘DP’ or ‘DS’ based on canonical markers (I) (Figure S3). tdTomato expression overlayed on uninjured fibroblast UMAP (J)

These scRNA‐seq data are the first to contain definitive neogenic dermal papilla from large regenerating wounds.^12^, ^13^ To investigate the contribution of Hic1 labelled cells to regenerating dermal papilla of hair follicles in WIHN, we utilized the scRNA‐seq strategy of Abbasi et al., 2020.^15^. Furthermore, the data were generated from spatially distinct regions of the wound. In short, four different 10× Genomics libraries were generated from large wounds at 14 days postwound (14dpw) (Figure 3A). Hic1CreERt2 x ROSA26‐tdTomato mice were treated on postnatal days 3 and 4 with tamoxifen and allowed to mature to postnatal day 28, which generated the expansion of Hic1 lineage fibroblasts in juvenile skin. At P28, the transgenic mice were wounded with a 1.5‐cm^2^ swound on the dorsal region of the skin. The large wound was harvested 14 days postwound (14dpw). Importantly, the wound was first surgically separated by cutting out the centre (LWC) and the periphery (LWP) (Figure 3A Step 2a‐b). Single‐cell preparations were generated of LWC and LWP, which were then flow sorted for live cells. This strategy generated four 10× Genomic libraries which were as follows: LWC tdTomato negative (LWC 14dpw Neg), LWC tdTomato positive (LWC 14dpw Pos), LWP tdTomato negative (LWP 14dpw Neg) and LWP tdTomato positive (LWP 14dpw Pos) (Figure 3A Step 4). This elegant scRNA‐seq approach provides us with an avenue to computationally test the potentiality of Hic1 lineage contribution to WIHN dermal papilla, and to ascertain which location and fibroblast subtype is the most similar to the regenerative dermal papilla of WIHN.

We investigated tdTomato expression within the scRNA‐seq data by realigning all libraries to a reference genome containing tdTomato sequence for reanalysis (Figure 3B). The newly generated UMAP contained all upper and lower fibroblast populations identified by Crabp1, Mest, Plac8 and Tgfbi expression (Figure 3B and data not shown). Next, we overlayed the four conditions defined by wound location and flow‐sorted tdTomato detection onto the new UMAP (Figure 3C). As in Figure 3B,C,E,F, the regenerating dermal papilla cluster consisted mainly (85%) of fibroblasts that were negative for tdTomato by flow cytometry (Figure 3C). In fact, only 3% of the neogenic dermal papilla originated from LWC 14dpw Pos flow‐sorted library. We also verified that regenerating dermal papilla from LWC 14dpw Neg was also negative for tdTomato transcripts (Figure 3D). tdTomato transcripts were not detected in both regenerating dermal papilla and in some of the upper wound fibroblast populations in cluster 5 (41.45% from LWC and 56.96% LWP) (Figure 3D,E). We infer that large wound hair follicle neogenesis might arise from the upper wound papillary fibroblast populations that may not express tdTomato and that the regenerative potential is similar between the periphery and centre of the large wound.

### 3.4 Understanding why neogenic dermal papilla are tdTomato negative within scRNA‐seq data

Abbasi et al. utilized the Hic1CreERT2‐tdTomato transgenic mouse model to lineage trace dermal fibroblast contribution of Hic1 during the large wound‐healing process. The histological analysis of the lineage tracing experiments revealed tdTomato contribution to all fibroblast populations in the wound bed including the regenerating hair follicle dermal papillae. However, the full analysis of the complimentary scRNA‐seq experiment performed in Abbasi et al. does not fully corroborate the histological findings (Figure 3C,D). Here, we proposed the following explanation to address this discrepancy.

Explanation 1): One explanation for the contradictory results is that the histology and the scRNA‐seq experiment had different labelling efficiencies marking different fibroblast populations. The labelling efficiencies of CreERT2 transgenic mouse models are influenced by a variety of factors including spatial/temporal expression of the transgene CreERT2, dosage of tamoxifen and recombination efficiency of the reporter line (ROSA‐tdTomato). Consequently, changes in the administration of tamoxifen by time, dosage and personnel can drastically change the specificity of CreERT labelling.^23^, ^24^, ^25^ Abbasi et al. reported that labelling dermal cells at P3/4 with the Hic1CreERT2‐tdTomato transgenic mouse model has the potential to label the papillary and reticular dermis, with a preference for reticular fibroblasts. Furthermore, the papillary and reticular dermis has different roles in supporting WIHN. Papillary fibroblasts in wounds can differentiate into dermal papillae, while reticular fibroblasts do not directly support hair follicle regeneration. Consequently, different labelling efficiencies of these fibroblast subtypes will influence the lineage tracing outcome in large wounds. In Figure 3F,G, we proposed the schematic presentation of two scenarios that differ by the percentage of papillary fibroblasts labelled by Hic1CreERT2. The histological experiments might have labelled higher percentages of papillary fibroblasts, while the labelling in the scRNA‐seq experiments is more specific to the reticular lineage. Due to the low number of labelled papillary fibroblasts, the scRNA‐seq experiments would result in the absence of tdTomato in regenerate dermal papilla.

Explanation 2): Another potential explanation is an anomaly whereby reporter expression is specifically downregulated in flow‐sorted reporter‐positive DP cells (but not other fibroblast subtypes). As a consequence, reporter genes may succumb to dropout in DP with droplet‐based single‐cell barcoding platforms (Figure 3H and Figure S3). Uninjured skin processed using 10xV2 confirms tdTomato is similarly downregulated and dropped out as only 17% of DP are tdTomato+ve (compared to 65% of tdTomato+ve DS and 76% of tdTomato+ve interfollicular fibroblasts; Figure S1N,O). By flow sorting‐enrichment of tdTomato‐high subset (to limit contamination from tdTomato‐ve fraction), Abbasi et al. may have inadvertently excluded tdTomato‐low DP. This provides an explanation for tdTomato+ upper LWP identified as the direct source of tdTomato‐low/negative neogenic DP (Figure 3F). Furthermore, we present two additional experiments that independently show similar results from a reanalysis of flow‐sorted alpha‐SMACreERT2‐RosaeYFP+ve hair follicle mesenchyme from uninjured skin (Figure S3).^26^ This additional explanation highlights the need for additional work to understand reporter gene expression in lineage tracing experiments with droplet‐based scRNA‐seq studies.

### 3.5 Upper wound fibroblasts from the wound periphery are predicted to have regenerative competence

**Figure 4:**
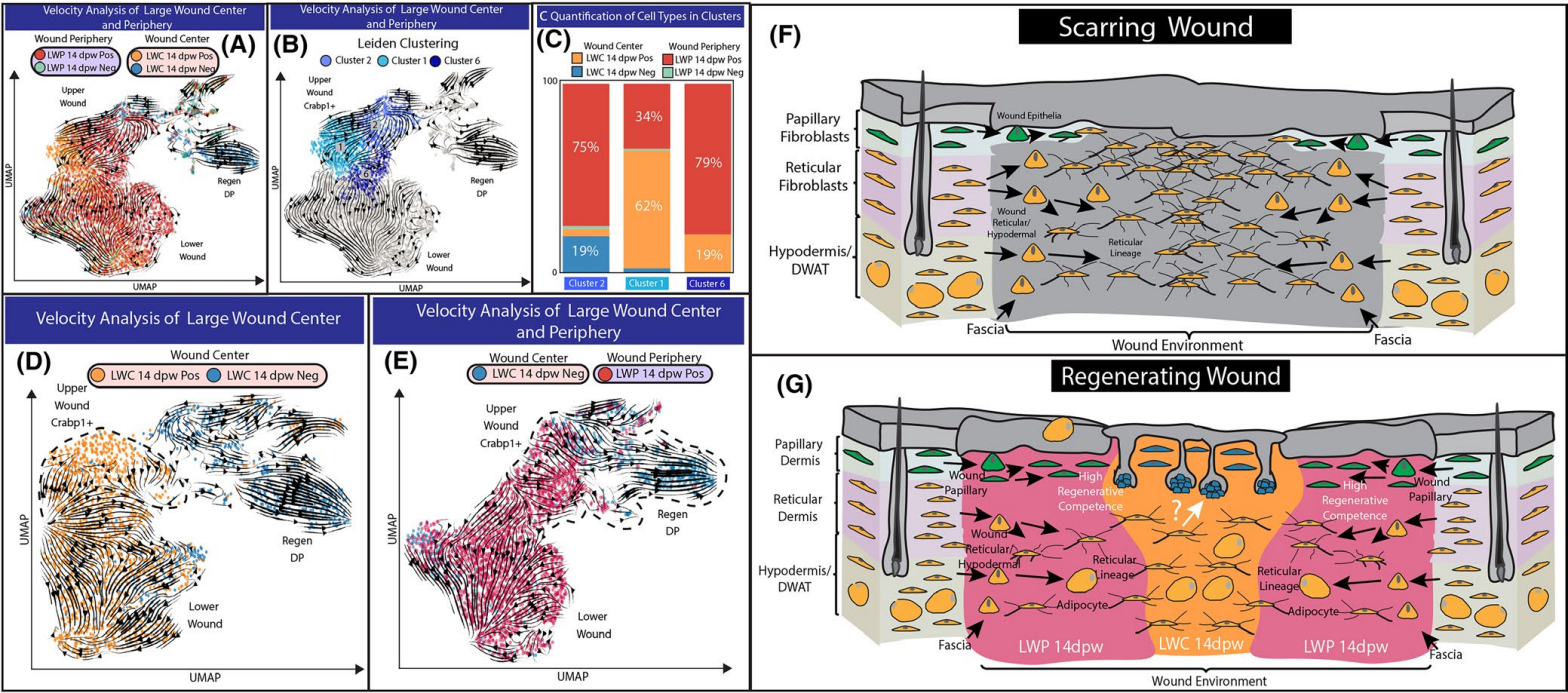
Computational test to identify the identify regeneration‐competent fibroblasts in large wounds. (A) Overlaying RNA velocity analysis in the context of four 10× genomics libraries generated for large wound periphery 14dpw tdTomato positive (LWP 14dpw Pos), large wound periphery 14dpw tdTomato negative (LWP 14dpw Neg), large wound centre 14dpw tdTomato Pos (LWC 14dpw Pos) and large wound centre 14dpw tdTomato negative (LWC 14dpw Neg). (B) Leiden clustering reveals three distinct clusters of Crabp1 upper wound fibroblasts. (C) Quantification of the number of cells from 10× genomic libraries represented in Leiden clusters. (D‐E) Computational test to determine the origin of the regenerative fibroblast and dermal papilla. (D) Velocity analysis of LWC 14dpw Pos and LWC 14dpw Neg libraries. (E) Velocity analysis of LWC 14dpw Neg and LWP 14dpw Pos libraries. (F) Proposed model for fibroblast heterogeneity in small scarring wounds. Papillary fibroblasts are green, while reticular/hypodermal/DWAT fibroblasts are yellow. (G) Proposed model for the contribution of different fibroblasts subtypes in large wounds

The scRNA‐seq approach from Abbasi et al., utilizing spatially derived 10× genomic libraries, provides an opportunity to investigate the effects of the spatial environments on fibroblast populations during wound healing. Here, we specifically test the four libraries of large wound fibroblasts to determine which cell populations exhibited the greatest competence to differentiate into dermal papillae (Figure 4A‐E). The clustering of cells on the UMAP analysis of fibroblast populations was done based on the similarities of their transcriptomes. Our UMAP analysis revealed three main populations of fibroblasts from all four libraries of large wounds: the lower wound fibroblasts expressing Mest/Plac8, the upper wound fibroblasts expressing Crabp1 and the neogenic DP expressing Rspo3/Corin (Figure 1G; Figure 4A,B). RNA velocity analysis depicted two distinct trajectories within the large wound fibroblast populations, where they diverged into either upper wound fibroblasts or lower wound fibroblasts. Due to the close association between upper wound fibroblasts and neogenic DP, we hypothesize that the regenerated DP in large wound arises from a population of upper wound fibroblasts.

To further study this sub‐population, we quantified the contribution of each library to each of the three Leiden clusters identified as upper wound fibroblasts (Figure 4C). Cluster 1 mainly composed of LWC tdTomato‐positive cells (79%) and was projected to become cluster 2, where 75% of the cells were LWP tdTomato positive cells. However, the RNA velocity trajectory of upper wound fibroblasts was disconnected from the neogenic DP when cells from all four libraries were included (Figure 4A‐C). Given the over‐representation of peripheral fibroblasts in cluster 2 (which was closest to the neogenic DP), we hypothesize that there might be distinct differences between upper wound fibroblasts from wound periphery and centre. We next performed a computational test using different conditions/libraries of large wound fibroblasts (Figure 4D,E). Since the neogenic DP were almost exclusively derived from the LWC tdTomato‐negative cells, we wanted to evaluate their relationship with cells from LWC tdTomato positive and LWP tdTomato positive. Our analysis suggested that the LWC tdTomato positive was not associated with the neogenic DP, as seen by the proximity of cells on UMAP (Figure 4D). RNA velocity also did not predict a trajectory of LWC‐positive upper wound fibroblasts towards the neogenic DP (Table S3). In contrast, the upper wound fibroblasts from the periphery clustered closely with the neogenic DP, and RNA velocity analysis predicted a continuous trajectory from peripheral upper wound fibroblasts towards the centre neogenic DP (Table S2). Additionally, we have also identified a population of LWC tdTomato‐negative cells that were closely associated with the neogenic DP (Figure 4D). These fibroblasts are most likely the source of the regenerating DP at the wound centre. Interestingly, the LWP upper wound fibroblasts also directly overlapped with this population, indicating their similarities in transcriptomic profiles (Figure 4E). It is important to note that inferences drawn from gene expression analysis may not capture the multi‐tiered regulatory dynamics that were highlighted in Abbasi et al., which may ultimately determine fibroblast function. Nevertheless, based on this analysis, we inferred that the population of peripheral upper wound fibroblasts exhibit competence to support regeneration of DP similar to the upper wound centre fibroblasts.

## 4 DISCUSSION

The rediscovery of hair follicle regeneration in large wounds in murine skin (a.k.a. WIHN) combined with the application of modern transgenic technology has provided for an essential model to dissect the cellular and molecular mechanisms required to support functional skin regeneration.^10^, ^11^, ^12^, ^13^, ^27^ The WIHN model system has been utilized in studies to reveal the importance of epidermal stem cell differentiation during wound repair and regeneration. However, one of the critical questions regarding the origins of the regenerative dermal papilla and fibroblasts that support WIHN remains unanswered.

Recently, Abbasi et al. report that Hic1 expressing fibroblasts labelled at P3/4 in both the papillary and reticular dermis give rise to the regenerative fibroblast population, including the regenerating dermal papilla.^15^ The conclusions were largely based on histological analysis of Hic1CreERt2‐tdTomato lineage tracing in which neogenic DP are reconstituted by tdTomato expressing cells. However, it is unclear whether the scRNA‐seq experiments performed in parallel corroborate these histological data. Here, our reanalysis suggests that Hic1 labelled reticular fibroblasts may not contribute to the regenerating dermis of WIHN.

Our reanalysis of the scRNA‐seq data sets revealed that the regenerating dermal papilla in the centre of the wound was negative or only sparsely expressed tdTomato at both transcript and protein levels (Figure 4B,C). In addition, tdTomato expression was also absent in up to half of the upper wound fibroblasts from both the central and periphery regions (Figure 4C). We present several plausible explanations that reconcile this discrepancy. One explanation for the differences in tdTomato detection between histological wound‐healing experiments in Abbasi et al. and our reanalysis of the scRNA‐seq experiments suggests varied efficiencies of Hic1CreERt2 labelling by tamoxifen injections between the two independent experiments performed. For example, the tdTomato expression found in histological analysis of regenerating WIHN may have arisen from increased papillary labelling, while other experiments preferentially labelled only the reticular/hypodermal layers (Figure 3F,G). Consequently, our analysis indicates that further characterization is required to better ascertain the Hic1CreERT2 specificity within fibroblast lineages and how this may be influenced by methods of tamoxifen administration. In support of this idea, Abbasi et al. show that Hic1CreERt2 could label at least 25% of papillary fibroblasts. Another equally plausible explanation is CMV‐driven reporters are downregulated and exhibit preferential dropout in DP cells when processed using droplet‐based single‐cell platforms (Figure 3H and Figure S3). Importantly, this downregulation might reflect the distinct functional state of specialized DP cells. Based on these caveats, ascribing definitive lineage origins of neogenic DPs based on the absence of reporter expression will require further study.

That said, it is also important to appreciate that cells in wound centre are the last to arrive and, unlike LWP upper fibroblasts, are still in an alpha‐SMA+ activated state. Although LWP upper is the predicted source of neogenic DP at day 14 postwound, LWC upper comprising Hic1‐tdTomato+papillary fibroblasts would be expected to acquire similar neogenic competence upon resolution of this transitionary myofibroblast state. Indeed, velocity analyses corroborate this view as vector fields suggest active transition of LWC upper towards this neogenic competent state. We hypothesize that an identical assessment of neogenic competence at a later timepoint would reveal that LWC and LWP upper fibroblasts harbour an equivalent likelihood to generate neogenic DP. Additionally, it is worth noting that inferences drawn from gene expression analyses alone do not capture the multi‐tiered regulatory dynamics. As demonstrated in Abbasi et al., gene expression is only one mechanism underlying regenerative competence. Convergence of signalling pathways leading to changes in regulatory network activity ultimately drives the acquisition of regenerative competence. The fact that regenerative propensity of large wounds (indicated by the number of neogenic HFs) remains amenable to pharmacological and genetic perturbations^28^, ^29^, ^30^, ^31^ supports the notion that fibroblasts in wound periphery harbour a latent degree of regenerative competence.

Our reanalysis of scRNA‐seq data from small and large wounds adds to the evidence for the role of restricted fibroblast lineages in wound repair and regeneration.^3^, ^10^, ^32^ Likewise, the reanalysed data support the hypothesis that the migration of papillary fibroblasts is a critical component for skin regeneration.^3^, ^33^ In conclusion, our in‐depth reanalysis of large wound scRNA‐seq data reveals that the field of fibroblast heterogeneity is an understudied area of research with great potential to uncover critical knowledge for understanding skin development, wound healing and regeneration.

## CONFLICT OF INTEREST

The authors have no conflict of interest.

## AUTHOR CONTRIBUTIONS

QMP processed, analysed, interpreted the data and co‐wrote manuscript. SS and JB provided the data, assisted in analysing and interpreting the data and co‐wrote the manuscript. RRD conceived of the project, assisted in analysing and interpreting the data, and co‐wrote the manuscript.

## Supporting information

Figure S1. Analysis of myofibroblast heterogeneity from SM22‐Control and SM22‐SmoM2 in wounded skin. (A) UMAP plot of subset fibroblasts colored by Leiden clusters. (B) UMAP plot of subset fibroblasts colored by conditions. (C) Quantification of cells contributed by each condition within fibroblasts subpopulations. (D) Marker genes expressions of 4 fibroblasts subpopulations projected on UMAP plot.

Figure S2. Upper wound myofibroblast from Hedgehog activated skin contribute to the formation of de‐novo dermal papilla in wounded skin. (A) RNA velocity projected as arrows on UMAP plot colored by Leiden clusters and conditions. (B) Presentation of the top 5 Velocity genes for each fibroblasts subpopulation. (C‐F) Projection of top 5 genes Velocity on UMAP plot. (G) Pathways with highest number of Velocity genes from Panther analysis. (H) List of Velocity genes in the top pathways. (I) Velocity of DP genes presented on UMAP plot.

Figure S3. CMV promoter‐driven reporter expression is downregulated and exhibits preferential drop‐out in DP cells. (A‐E) Reanalysis of flow‐sorted αSMACreERT2:RosaeYFP+ve HF mesenchyme (Shin et al., 2020) barcoded using 10× V2 chemistry reveals downregulation and drop‐out of eYFP transcripts in DP. (A) Experimental design for fate‐mapping, flow sorting, and single‐cell transcriptomic experiments using αSMACreERT2:RosaeYFP mice. (B) UMAP of HF fibroblasts subclustered using original Louvain algorithm and grouped as “DP” or “DS” based on canonical markers presented in Panel c. (C) Expression of DS (Cd200, Acta2) and DP (Rspo3, Lef1) markers. (D) CMV‐driven eYFP expression visualized as feature and density plots. DP/DS boundaries are marked by dashed lines. (E) Scatter plots displaying negative correlations between Rspo3 versus eYFP and Lef1 versus eYFP expression. (F‐J) Reanalysis of independently generated flow‐sorted SMACreERT2:RosaeYFP+ve hair follicle mesenchyme (Shin et al., 2020) barcoded using 10× V3 chemistry also reveals downregulation of eYFP transcripts in DP. (F) Identical experimental design as described in Panel A with the exception of barcoding performed using 10× V3 chemistry. (G) UMAP projection of HF fibroblasts subclustered using original louvain algorithm and grouped as “DP” or “DS” based on canonical markers shown in Panel h. (H) Expression of DS (Cd200, Acta2) and DP (Rspo3, Lef1) markers. (I) CMV‐driven eYFP expression visualized as feature and density plots. DP/DS boundaries are marked by dashed lines. (J) Scatter plots displaying negative correlations between Rspo3 versus eYFP and Lef1 versus eYFP expression. (K‐O) Reanalysis of flow‐sorted Hic1CreERT2:RosatdTomato+ve follicular and interfollicular fibroblasts (Abbasi et al., 2020) barcoded using 10× V2 chemistry reveals downregulation and drop‐out of tdTomato transcripts in DP. (K) Experimental design for fate‐mapping, flow sorted, and single‐cell transcriptomic experiments using Hic1CreERT2:RosatdTomato mice. (L) UMAP projection of fibroblasts subclustered using original louvain algorithm and grouped as “Interfollicular Fibroblasts”, “DP”, or “DS” based on canonical markers shown in Panel m. (M) Expression of pan‐ dermal fibroblast (Pdgfra), DS (Cd200, Acta2) and DP (Rspo3, Lef1) markers. (N) CMV‐driven eYFP expression visualized as feature and density plots. DP/DS boundaries are marked by dashed lines. (O) Ordered ridge plot comparing tdTomato expression across fibroblast subsets.

Table S1. List of 362 velocity genes.

Table S2. Periphery test velocity genes Figure 4E.

Table S3. Center test velocity genes from Figure 4D.
