# Supplementary figures and images for: Single‐cell transcriptomic analysis of small and large wounds reveals the distinct spatial organization of regenerative fibroblasts
Source: Exp Dermatol. 2020 Dec 7;30(1):92–101. doi: 10.1111/exd.14244 (PMC7839523; doi:10.1111/exd.14244)

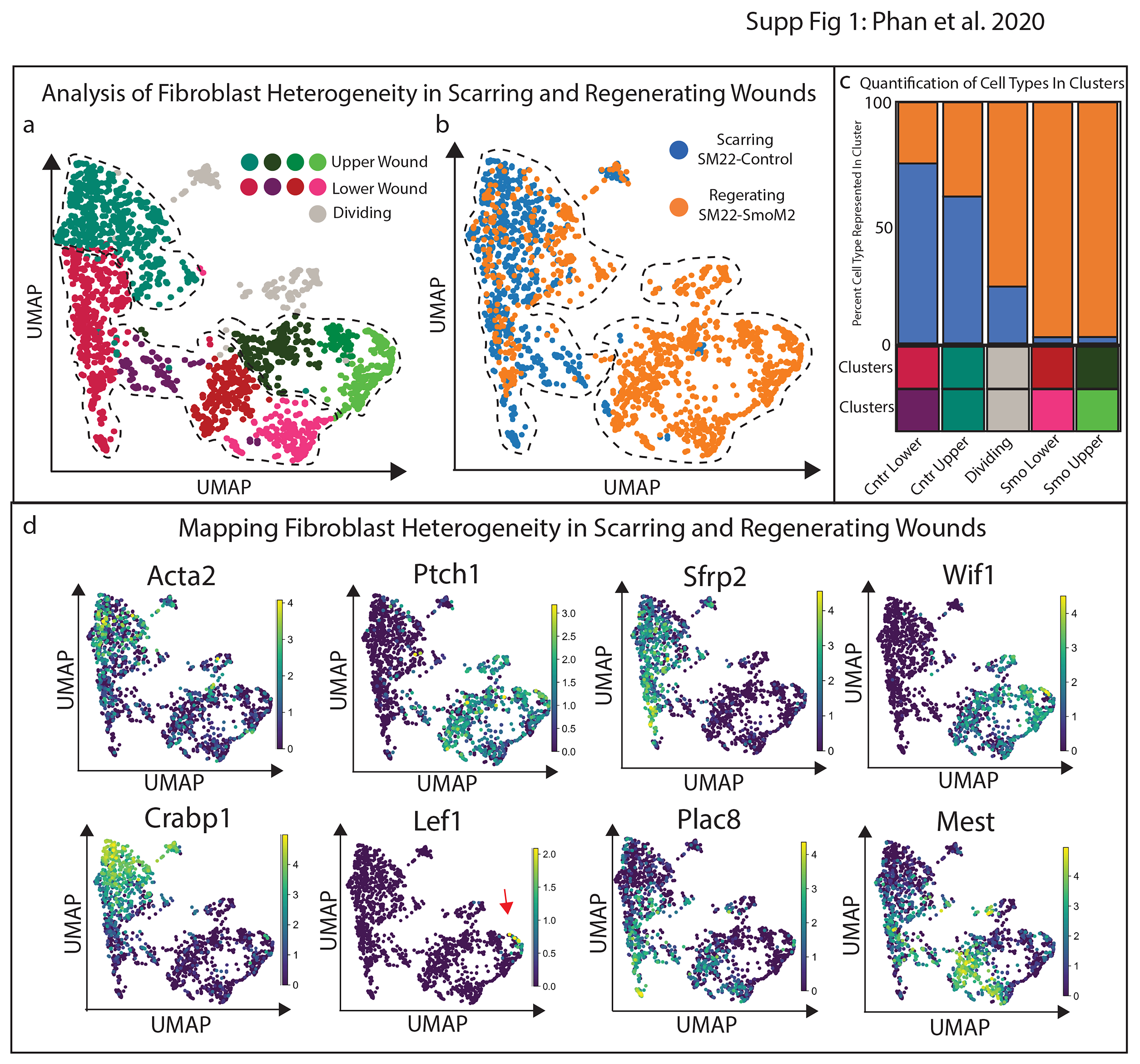

Supplement: Supplementary file 1 — Figure S1. Analysis of myofibroblast heterogeneity from SM22‐Control and SM22‐SmoM2 in wounded skin. (A) UMAP plot of subset fibroblasts colored by Leiden clusters. (B) UMAP plot of subset fibroblasts colored by conditions. (C) Quantification of cells contributed by each condition within fibroblasts subpopulations. (D) Marker genes expressions of 4 fibroblasts subpopulations projected on UMAP plot. [file EXD-30-92-s001.jpg]

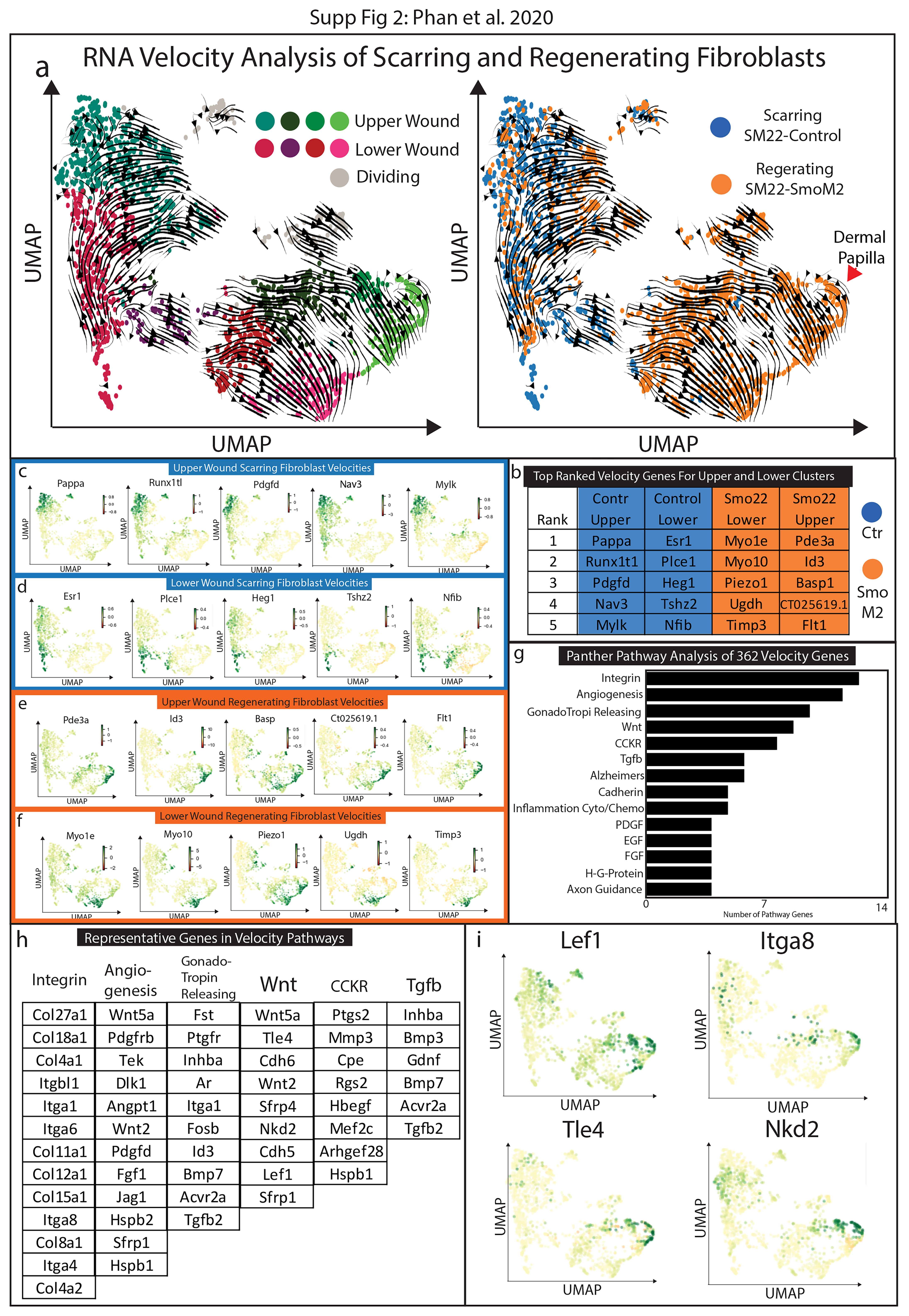

Supplement: Supplementary file 2 — Figure S2. Upper wound myofibroblast from Hedgehog activated skin contribute to the formation of de‐novo dermal papilla in wounded skin. (A) RNA velocity projected as arrows on UMAP plot colored by Leiden clusters and conditions. (B) Presentation of the top 5 Velocity genes for each fibroblasts subpopulation. (C‐F) Projection of top 5 genes Velocity on UMAP plot. (G) Pathways with highest number of Velocity genes from Panther analysis. (H) List of Velocity genes in the top pathways. (I) Velocity of DP genes presented on UMAP plot. [file EXD-30-92-s002.jpg]

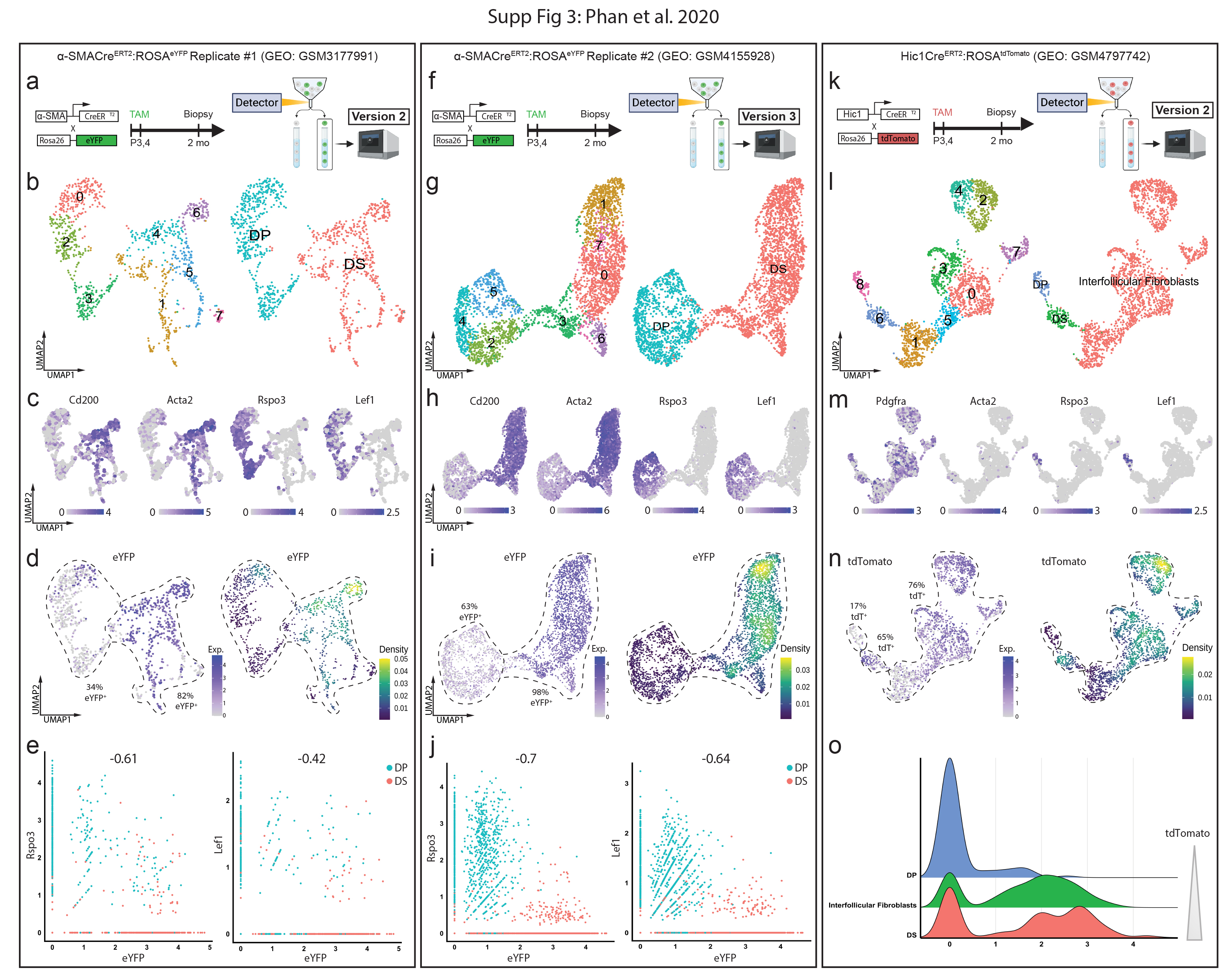

Supplement: Supplementary file 3 — Figure S3. CMV promoter‐driven reporter expression is downregulated and exhibits preferential drop‐out in DP cells. (A‐E) Reanalysis of flow‐sorted αSMACreERT2:RosaeYFP+ve HF mesenchyme (Shin et al., 2020) barcoded using 10× V2 chemistry reveals downregulation and drop‐out of eYFP transcripts in DP. (A) Experimental design for fate‐mapping, flow sorting, and single‐cell transcriptomic experiments using αSMACreERT2:RosaeYFP mice. (B) UMAP of HF fibroblasts subclustered using original Louvain algorithm and grouped as “DP” or “DS” based on canonical markers presented in Panel c. (C) Expression of DS (Cd200, Acta2) and DP (Rspo3, Lef1) markers. (D) CMV‐driven eYFP expression visualized as feature and density plots. DP/DS boundaries are marked by dashed lines. (E) Scatter plots displaying negative correlations between Rspo3 versus eYFP and Lef1 versus eYFP expression. (F‐J) Reanalysis of independently generated flow‐sorted SMACreERT2:RosaeYFP+ve hair follicle mesenchyme (Shin et al., 2020) barcoded using 10× V3 chemistry also reveals downregulation of eYFP transcripts in DP. (F) Identical experimental design as described in Panel A with the exception of barcoding performed using 10× V3 chemistry. (G) UMAP projection of HF fibroblasts subclustered using original louvain algorithm and grouped as “DP” or “DS” based on canonical markers shown in Panel h. (H) Expression of DS (Cd200, Acta2) and DP (Rspo3, Lef1) markers. (I) CMV‐driven eYFP expression visualized as feature and density plots. DP/DS boundaries are marked by dashed lines. (J) Scatter plots displaying negative correlations between Rspo3 versus eYFP and Lef1 versus eYFP expression. (K‐O) Reanalysis of flow‐sorted Hic1CreERT2:RosatdTomato+ve follicular and interfollicular fibroblasts (Abbasi et al., 2020) barcoded using 10× V2 chemistry reveals downregulation and drop‐out of tdTomato transcripts in DP. (K) Experimental design for fate‐mapping, flow sorted, and single‐cell transcriptomic experiments using Hic1CreERT2 [file EXD-30-92-s003.jpg]
